# Public funds, public safety: a scoping review of government spending decisions and their impact on interpersonal violence and suicide

**DOI:** 10.1186/s40621-025-00624-7

**Published:** 2025-12-29

**Authors:** Julia J. Lund, Julia P. Schleimer, Paul M. Reeping, Veronica A. Pear

**Affiliations:** Department of Emergency Medicine, Centers for Violence Prevention, University of California Davis, Sacramento, CA USA

**Keywords:** Violence prevention, Suicide prevention, Social determinants of health, Government spending

## Abstract

**Objectives:**

To conduct a scoping review of research on government spending and violence.

**Methods:**

We searched nine databases for peer-reviewed publications evaluating the association between government spending on public goods and services and (other- or self-directed) violent physical injury or death in the United States.

**Results:**

Of 5,734 screened articles, 33 met the inclusion criteria. Over one-third of studies were published in the last ten years (*n* = 13). Studies most commonly evaluated spending at the state (*n* = 19), county (*n* = 5), and city (*n* = 5) levels. Studies examined spending on social welfare (*n* = 21), health (*n* = 12), education (*n* = 9), law enforcement (*n* = 8), and/or community development (*n* = 2). Outcomes were homicide/assault (*n* = 28) and suicide (*n* = 17). All studies were ecologic, 24 were serial cross-sectional, and most (*n* = 28) made attempts to control for confounding. Findings were mixed, but studies of social welfare, health, and education most commonly found that increased spending was significantly associated with reductions in violence.

**Conclusions:**

While research has varied somewhat in methodology and findings, results of this scoping review indicate that government investments in supportive services may be promising structural interventions to prevent violence and promote safety and health.

## Background

The burden of violence in the United States (US) is staggering and far outpaces the burden in similarly large, wealthy countries [1]. In the past decade, there have been more than 700,000 violence-related deaths (homicides and suicides) in the US and many more nonfatal violent injuries [2]. In 2020, firearms became the leading cause of death among children in the US, and firearm homicide rates reached heights unseen in 25 years [3]. The full impact of violence extends beyond injuries and deaths alone, with consequences reverberating throughout neighborhoods and across generations [4].

In the US, interpersonal violence and suicide are deeply intertwined with social and structural determinants of health, which encompass “the conditions in which people are born, grow, live, work, and age, and the systems and forces shaping those conditions” [5]. Exposure to adverse social determinants of health – such as poverty, pollution, food insecurity, lack of education and employment opportunities, insufficient healthcare, and unstable housing – increase risk of homicide [6] and suicide [7] through numerous pathways, including stress, deprivation, desperation, and trauma. Thus, the unequal distribution of these and other adverse social determinants of health has resulted in the unequal distribution of violence (both interpersonal and self-directed) along the lines of race, socioeconomic class, gender, and other dimensions of marginalization [6, 8]. For instance, homicide disproportionately burdens Black and Brown communities; firearm homicide has been the leading cause of death for Black males aged 15 to 34 for over 30 years and is the second-leading cause of death for Latino males and Black females aged 15 to 24 [2]. Suicide rates are highest among American Indian/Alaska Native individuals and white men, with increased risk for those identifying as LGBTQ (lesbian, gay, bisexual, transgender, or queer), those with disabilities, veterans, and residents of rural areas [9]. Such inequities in violence and the social conditions that fuel it stem from and reinforce structural violence, or violence resulting from oppressive societal relations and power structures [10].

Public investment decisions may be key structural drivers of interpersonal violence and suicide because they directly influence the availability and quality of certain social determinants of health that can either sustain or disrupt cycles of violence. While the connection between historic underinvestment and contemporary violence has been increasingly researched [11], there remains little consensus of how modifying government budget allocation, particularly by investing more to address the root causes of violence, can impact violence-related outcomes. Governments may lack control over the amount of funds in their budgets, but they hold significant agency over how those budgets are spent; such choices are likely to have profound implications across many health outcomes, including violence [12–14].

Despite a wealth of research and first-hand testimony from those impacted by interpersonal violence and suicide that establishes these crises as a symptoms of systemic injustice [6, 10, 15, 16], government approaches to promoting public safety and health often over-prioritize reactionary and punitive methods that do not address, and often exacerbate, these underlying drivers [12]. For instance, a recently conducted review of municipal spending trends found that, over the past 50 years, city budgets have steadily reduced their investment in social services by 12%, while increasing investment in police budgets by 19% [12]. In response to homicide and community violence, resources are often disproportionately allocated to police and prisons versus the essential needs of affected communities and individuals. In response to suicide, self-harm, and other behavioral health emergencies, crisis response systems often lack a trauma-informed approach, criminalizing those in need instead of connecting them to necessary supports [17], while funding for mental health services remains insufficient relative to the scale of the problem [18]. This prevailing paradigm fails to address the underlying upstream causes of interpersonal violence and suicide and disregards the perspectives of those most impacted, who have long suggested governments focus on addressing underlying social conditions to prevent violence [19–21].

In recent years, there have been several innovative safety-related budget proposals and decisions across the country at various levels of government. For instance, in 2021, the federal government proposed $5 billion dollars to fund community-led approaches to building peace [22, 23] and in 2022, it passed the first federal gun safety legislation in nearly 30 years, which included provisions for federal agencies to direct funding to community violence and crisis intervention programs [24]. In addition, some city governments distributed a portion of the national American Rescue Plan Act funds to support community-based violence prevention strategies [25], and a growing number of communities began implementing alternative models to traditional policing, like having social/mental health workers responding to certain 911 calls [26]. Relatedly, an increasing number of localities have begun implementing the democratic process of participatory budgeting, which involves directly engaging community members in deciding how a portion of public funds should be allocated to address local needs and priorities [27]. This momentum has been facilitated, in part, by the growing national conversations about, and collective demand to address, inequities in health and safety and insufficiencies in our criminal legal system. These acknowledgments were stirred by events in 2020 and 2021 that laid bare long-standing injustices, including the inequitable health and economic repercussions of the COVID-19 pandemic [28, 29] and the murder of George Floyd and subsequent protests over police brutality and racial injustice [30]. With the Trump administration now altering federal priorities – including removing the federal Office of Gun Violence Prevention and defunding public health, community violence intervention, and equity-related initiatives [31] – it is particularly critical to deepen our understanding of the pathways between government spending and public safety.

Amid this shifting political and financial landscape, which has seen both growing motivation for creative budget decisions and recent setbacks, it is essential to understand how government spending decisions can most effectively and sustainably prevent interpersonal violence and suicide. This review critically examines the academic literature on the relationship between interpersonal violence and suicide and government spending on several upstream causes (including social welfare, health, community development, and education) and on more traditionally punitive approaches (law enforcement spending). We aim to answer if and how researchers have approached these topics, examine the extent to which findings point to consensus, and explore gaps in the literature to inform future research.

While scholars have previously reviewed the literature on the impacts of social policies and programs on firearm violence [14] and suicide [32], no reviews, to our knowledge, have examined both interpersonal and self-directed violence regardless of injury mechanism, nor comprehensively examined government spending at a macro-level as a structural determinant of violence. By focusing on major budget categories, this review complements program- and policy-level research by examining how resources are allocated rather than how programs are implemented, highlighting patterns across investment, providing insight into structural drivers of interpersonal violence and suicide, and enabling consideration of the structural context in which programs and policies operate. Due to the broad and nascent nature of our research questions, scoping review methodology is best positioned to characterize this body of research and identify what is known and what is missing.

## Methods

To ensure rigor and transparency, we used a 5-stage approach for scoping reviews [33] with reporting guided by the Preferred Reporting Items for Systematic reviews and Meta-Analyses Extension for Scoping Reviews (PRISMA-ScR) [34].

We began by identifying the research question (Stage 1) through a preliminary, unstructured literature search and discussion among all authors. We identified three main questions to guide our review: (1) to what extent and how has the relationship between government spending and violence (including interpersonal or self-directed) been studied? (2) is government spending associated with violence? and (3) which types of government spending, if any, do studies suggest are most protective against violence?

To identify studies (Stage 2), on March 19, 2025, we searched nine databases relevant to the subject matter, selected in collaboration with a University of California, Davis librarian: PubMed, Black Studies Center, EconLit, ERIC, PAIS Index, Sociological Abstracts, Social Services Abstracts, Scopus, and Web of Science Core Collection. Because our review focused on peer-reviewed academic studies, we did not include grey literature sources in our search. Search terms, generated iteratively in collaboration with the librarian and all authors, included a combination of terms related to governmental entities (e.g. “state” OR “county”), budget allocation (e.g. “investment” OR “spending”), spending type (e.g. “education” OR “health”), and violence (e.g. “violence” OR “suicide”). Specific search terms are in Appendix A.

Inclusion criteria for study selection (Stage 3) were iteratively developed by all authors. To be included, a study had to be a full-length, original empirical article; peer-reviewed; with a study population in the US; and with an exposure of government spending, in dollars (not a relative change over time), in one or more of the following areas: social welfare, health (including mental health or public health), community development (including housing or parks and recreation), education, or law enforcement. We restricted inclusion to studies assessing government budgets broadly, rather than allocation to specific programs or services (i.e., Medicaid spending, or Community Oriented Policing Services grants). The outcome of the study had to be a measure of violent crime, injury, or death, defined as physical interpersonal or self-directed harm (fatal or non-fatal). We required that studies provide a point estimate but did not impose any restrictions on study design or analysis type.

A preliminary review was done by the first author to exclude non-US and non-peer reviewed articles. The remainder of articles were divided equally among all authors, who reviewed titles and abstracts for inclusion/exclusion criteria. Questions were reviewed as a group, with decisions finalized via consensus. Records that remained after preliminary title and abstract review were then downloaded, and the full text was reviewed by two authors. Decisions on final inclusion were made collectively by all authors.

We then charted data from included studies (Stage 4), extracting the following elements into a data matrix: authors’ names, first author’s academic department or primary area of work as noted in their affiliation, year of publication, study period, unit of exposure (level of government), government spending category, violence-related outcome of interest, study design, analytic method, and the main findings and conclusions.

Finally, we collated and summarized results (Stage 5) by identifying patterns and themes from the content of the data matrix. While the comparability of study designs and research contexts were too low to warrant a meta-analysis of results [35], we produced tables and charts describing the literature.

## Results

**Fig. 1:**
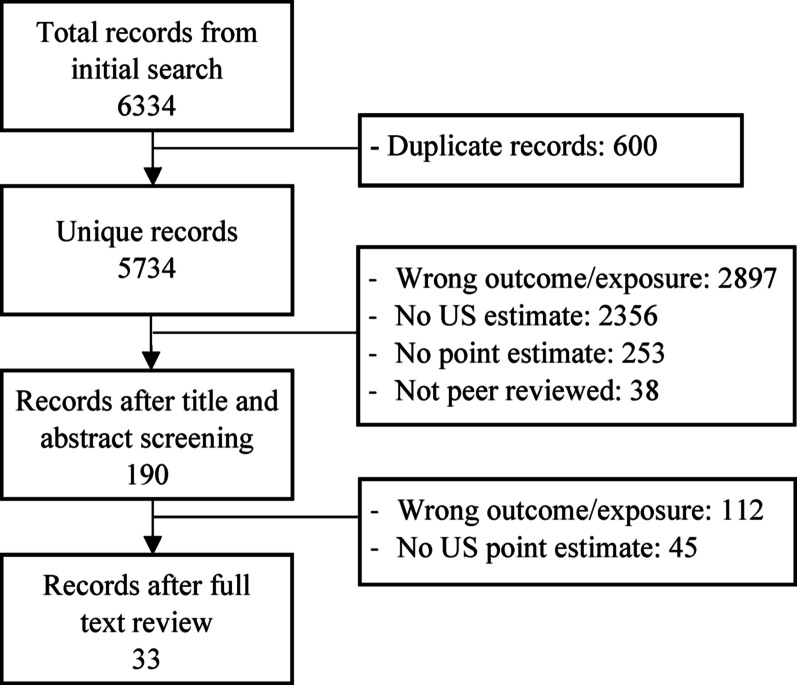
Record review process

After deduplication, we identified 5,734 unique records (Fig. 1). We excluded 5,544 articles following title and abstract review and 157 after full text review. Ultimately, 33 articles [36–68] met inclusion criteria (Table 1). These papers were mostly published after 2000 (*n* = 25) by researchers with professional affiliations in public health, sociology, criminology, or economics.

**Table 1:** Summary of included studies, sorted by spending category and violence outcome

Spending area	Violence outcome	Direction of association with increased spending, when significant^1^	Study setting and sample size	Study period	Study design	Analytic strategy (broadly)	Adjusted for any con-founders	Point estimate	First author (Publication year)	First author discipline
Social welfare	Homicide/assault	Negative	114 cities	1929–1940	Serial cross-sectional	Two-stage least-squares	Yes	1 standard deviation increase in relief spending per capita associated with 0.21 standard deviation lower homicide rate	Fishback, P. (2005)	Economics
Social welfare	Homicide/assault	Negative	406 counties	1990	Cross-sectional	Ordinary least squares	Yes	Significant negative effect of the amount of public assistance income per poor recipient on violence crime rates (beta = − 0.13) at the mean level of resource deprivation	Hannon, L. (1998)	Sociology
Social welfare	Homicide/assault	Negative	50 states	1994–2014	Serial cross-sectional	Linear regression with panel-corrected standard errors	Yes	An increase in welfare expenditures by 1% point is associated with a reduction in violent crime rate by 0.117% points	Hazra, D. (2022)	Economics
Social welfare	Homicide/assault	Negative	74,134 census tracts in 48 states	2005–2015	Serial cross-sectional	Negative binomial regression	Yes	$250 more per capita welfare spending associated with 12% reduction in firearm-related homicides 5 years later	Kim, D. (2019)	Public health
Social welfare	Homicide/assault	Negative	50 states + DC	2010–2017	Serial cross-sectional	Generalized estimating equations	Yes	Each additional $1000 spent by states on benefit programs (sum of spending on cash, housing, and in-kind assistance, low-income housing, child care assistance, EITC, and medical assistance programs) per person living in poverty was associated with a 7.7% decrease in maltreatment-related fatalities (including neglect, physical abuse, and sexual abuse) for children, and 4.0% decrease in substantiated reports of child maltreatment	Puls, H. (2021)	Public health
Social welfare	Homicide/assault	Negative	58 counties in California	1990–1998	Serial cross-sectional	Generalized method of moments	Yes	Average amount of money spent each year for persons on general relief inversely related to homicide rate per 100,000 people (beta = − 0.004)	Worrall, J. (2009)	Criminology and political science
Social welfare	Homicide/assault	Positive	454 counties	1990	Cross-sectional	Poisson regression	No	Homicide rates are on average roughly one-third lower (*r* = −.356) in urban counties where the average monthly cost of living-adjusted welfare payment per poor person is disbursed at low to moderate levels (quartiles 1–3) than where welfare payments are disbursed at high levels (quartile 4)	Maume, (2003)	Sociology and criminal justice
Social welfare	Homicide/assault	Null	50 states	1997–2006	Serial cross-sectional	Fixed-effects regression	Yes	Welfare support spending per person in poverty not significantly associated with violent crime rates	Brown, J. (2016)	Sociology
Social welfare	Homicide/assault	Null	National	1948–1985	Serial cross-sectional	Two-stage least-squares	Yes	The amount of relief spending (the sum of public spending on aid to families with dependent children, categorical public assistance benefits, and other direct relief) not significantly associated with homicide rates	Devine, J. (1988)	Sociology
Social welfare	Homicide/assault	Null	50 states	2015–2020	Serial cross-sectional	Fixed effects negative binomial regression	Yes	Per capita state spending on public welfare statistically significantly associated with no change in injurious shootings by police within the county. IRR = 1.00 (95% CI: 1.00, 1,00)	Ward, J. (2025)	Public Health
Social welfare	Homicide/assault	Null	58 counties in California	1990–1998	Serial cross-sectional	Two-way fixed-effects	Yes	Welfare spending (measured as per capita social service spending, per capita general relief, and per capita family assistance) not significantly associated with homicide or assault rates	Worrall, J. (2005)	Criminology and political science
Social welfare	Suicide	Negative	114 cities	1929–1940	Serial cross-sectional	Two-stage least-squares	Yes	1 standard deviation increase in relief spending per capita associated with 0.85 standard deviation lower suicide rate	Fishback, P. (2005)	Economics
Social welfare	Suicide	Negative	50 states	1990–2000	Serial cross-sectional	Ordinary least squares	Yes	Roughly $45 more per capita welfare spending in every state estimated to result in 3000 fewer suicides nationally per year	Flavin, P. (2009)	Political science
Social welfare	Suicide	Negative	47 states	1982–1997	Serial cross-sectional	System generalized method of moments estimation	Yes	0.01% point increase in the share of total budget allocated to welfare associated with 0.74% lower suicide rate after 1 year	Minoiu, C. (2008)	Economics
Social welfare	Suicide	Negative	50 states	1982	Cross-sectional	Correlation	Yes	Public welfare expenditure per capita correlated with lower suicide rates (*r* = −.45)	Zimmerman, S. (1988)	Social work
Social welfare	Suicide	Negative	50 states	1960, 1970, 1980, 1985, 1990	Cross-sectional	Multiple-regression	Yes	Public welfare spending per capita associated with lower suicide rates (beta = − 0.23)	Zimmerman, S. (1995)	Social work
Social welfare	Suicide	Negative	50 states	1960, 1970, 1980, 1985, 1990	Serial cross-sectional	Ordinary least squares	Yes	$1 increase in public welfare spending per capita associated with decrease in suicide rates by 0.004 per 100,000	Zimmerman, S. (2002)	Social work
Social welfare	Suicide	Null	48 states	1979–1987	Serial cross-sectional	Ordinary least squares with state and time fixed effects	Yes	No significant association between $250 more per capita welfare spending and suicide	Kim, D. (2016)	Public health
Social welfare	Suicide	Null	50 states	2000–2015	Serial cross-sectional	Two-way fixed-effects	Yes	Percent of state income allocated to public welfare expenditures not significantly associated with suicide rates	Rambotti, S. (2020)	Sociology
Social welfare	Suicide	Null	50 states + DC	1997–2005	Serial cross-sectional	System generalized method of moments estimation	Yes	Public welfare expenditures not significantly associated with suicide rates	Ross, J. (2012)	Economics
Social welfare	Suicide	Null	50 states	1982	Cross-sectional	Stepwise multiple-regression	Yes	State public welfare expenditure not significantly associated with suicide	Zimmerman, S. (1987)	Social work
Health	Homicide/assault	Negative	50 states	2005–2013	Serial cross-sectional	Two-stage least squares	Yes	$1 per capita increase in community-directed state mental health agency spending per capita associated with 1.5–1.6 fewer violent crimes per 100,000, and 0.36–0.44 fewer violent crimes with a firearm per 100,000	Palatucci, J. (2021)	Public health
Health	Homicide/assault	Null	100 counties, in North Carolina	2004–2006	Serial cross-sectional	Poisson regression	Yes	Null association between per capita funding for domestic violence services and count of intimate partner homicide rate	Madkour, A. (2011)	Public health
Health	Homicide/assault	Null	50 states	2002	Cross-sectional	Partial correlation	No	Per capita expenditures for mental health care services not significantly associated with firearm homicide	Price, J. (2009)	Public health
Health	Homicide/assault	Null	50 states	2015–2020	Serial cross-sectional	Fixed effects negative binomial regression	Yes	Per capita state spending on health statistically significantly associated with no change in injurious shootings by police within the county. IRR = 1.00 (95% CI: 1.00, 1,00)	Ward, J. (2025)	Public Health
Health	Suicide	Negative	50 states	2010, 2017	Cross-sectional	Correlation	No	Teen suicide rates decreased with increased state behavioral health spending (*r* = −.316)	Godshall (2024)	Social work
Health	Suicide	Negative	47 states	1982–1997	Serial cross-sectional	System generalized method of moments estimation	Yes	0.01% point increase in the share of the total budget allocated to health associated with 1.54% lower suicide rate after 1 year	Minoiu, C. (2008)	Economics
Health	Suicide	Negative	50 states	2002	Cross-sectional	Partial correlation	No	Firearm suicide rates decreased with increasing per capita expenditures for mental health care services (*r* = −.33)	Price, J. (2009)	Public health
Health	Suicide	Negative	50 states + DC	1997–2005	Serial cross-sectional	System generalized method of moments estimation	Yes	Public health expenditures per capita associated with lower suicide rates among males (beta = − 0.038), not significantly associated with female suicide rates	Ross, J. (2012)	Economics
Health	Suicide	Negative	50 states	1982	Cross-sectional	Correlation	Yes	Mental health expenditures per capita correlated with lower suicide rates (*r* = −.22)	Zimmerman, S. (1988)	Social work
Health	Suicide	Positive	48 states	1979–1987	Serial cross-sectional	Ordinary least squares with state and time fixed effects	Yes	$250 more per capita healthcare spending associated with 0.04% point increase in risk of death by suicide	Kim, D. (2016)	Public health
Health	Suicide	Null	50 states	1997	Cross-sectional	Descriptive	No	State hospital expenditure not significantly associated with suicide rates	Davis, G. (2002)	Psychiatry
Health	Suicide	Null	50 states	1960, 1970, 1980, 1984	Serial cross-sectional	Multiple-regression	Yes	Prior to 1984, spending for hospitals not associated with suicide. In 1984, state’s spending for hospitals inversely related to suicide rates (beta = 0.16)	Zimmerman, S. (1990)	Social work
Education	Homicide/assault	Negative	50 states	1994–2014	Serial cross-sectional	Linear regression with panel-corrected standard errors	Yes	1% point increase in educational expenditure associated with a 0.082% point reduction in violent crime rate	Hazra, D. (2022)	Economics
Education	Homicide/assault	Negative	74,134 census tracts in 48 states	2005–2015	Serial cross-sectional	Negative binomial regression	Yes	$250 more per capita education spending associated with 13% reduction in firearm-related homicides 5 years later	Kim, D. (2019)	Public health
Education	Homicide/assault	Negative	50 states	2002	Cross-sectional	Partial correlation	No	Firearm homicide rates decreased with increasing educational expenditures per pupil (*r* = −.28)	Price, J. (2009)	Public health
Education	Homicide/assault	Positive	13,454 school districts	1998–2018	Serial cross-sectional	Penalized maximum likelihood logistic regression	Yes	$1000 increase per student associated with 9.5% higher odds of experiencing a school shooting	Fridel, E. (2021)	Criminology
Education	Homicide/assault	Null	10,133 school districts	2003–2018	Serial cross-sectional	Linear mixed-effect regression	Yes	No significant association between a $1000 increase in school district spending per pupil on education and violent crime	Ades, J. (2021)	Psychiatry
Education	Homicide/assault	Null	454 counties	1990	Cross-sectional	Poisson regression	No	Null association between educational expenditures per pupil and homicide rates	Maume, (2003)	Sociology and criminal justice
Education	Suicide	Negative	50 states	2002	Cross-sectional	Partial correlation	No	Firearm suicide rates decreased with increasing educational expenditures per pupil (*r* = −.45)	Price, J. (2009)	Public health
Education	Suicide	Negative	50 states	1982	Cross-sectional	Correlation	Yes	Education expenditures per capita correlated with lower suicide rates (*r* = −.22)	Zimmerman, S. (1988)	Social work
Education	Suicide	Positive	48 states	1979–1987	Serial cross-sectional	Ordinary least squares with state and time fixed effects	Yes	$250 more per capita education spending associated with 0.04% point increase in risk of death by suicide	Kim, D. (2016)	Public health
Education	Suicide	Null	50 states	2010, 2017	Cross-sectional	Correlation	No	Teen suicide rates not significantly associated with per student educational expenditure	Godshall (2024)	Social work
Community development	Homicide/assault	Negative	85 cities	1990–2001	Serial cross-sectional	Two-way, fixed-effect panel model	Yes	1$ increase per resident on community development associated with 0.58 fewer violent crime incidents; $1 increase per resident on park and recreational facilities not significantly associated with violent crime	Ren, L. (2008)	Criminology
Law enforcement	Homicide/assault	Negative	85 cities	1990–2001	Serial cross-sectional	Two-way, fixed-effect panel model	Yes	$1 increase per resident on police expenditure associated with 2 fewer violent crimes per 100,000	Ren, L. (2008)	Criminology
Law enforcement	Homicide/assault	Negative	119 cities	1960	Cross-sectional	Two-stage least squares	Yes	Increases in police expenditure per capita associated with lower crime rates (beta = − 0.117)	Swimmer, G. (1974)	Economics
Law enforcement	Homicide/assault	Null	2 cities (Boston and Philadelphia)	2015–2020	Serial cross-sectional	Panel linear fixed-effects regression	Yes	Police budget not significantly associated with shootings or firearm homicide	Hatchimonji, J. (2023)	Emergency surgery
Law enforcement	Homicide/assault	Null	50 states	1994–2014	Serial cross-sectional	Linear regression with panel-corrected standard errors	Yes	No significant association between police expenditure and violent crime rate	Hazra, D. (2022)	Economics
Law enforcement	Homicide/assault	Null	74,134 census tracts in 48 states	2005–2015	Serial cross-sectional	Negative binomial regression	Yes	Null association between $250 more per capita police spending and homicide rates	Kim, D. (2019)	Public health
Law enforcement	Homicide/assault	Null	50 states	2015–2020	Serial cross-sectional	Fixed effects negative binomial regression	Yes	Per capita state spending on police not statistically significantly associated with injurious shootings by police within the county	Ward, J. (2025)	Public Health
Law enforcement	Homicide/assault	Null	21 cities	1960, 1970	Serial cross-sectional	Multiple correlation	Yes	Neither the per capita police budget nor the ratio of the police budget to the total city budget account for a significant amount of the variation in violent crime rates	Wellford, C. (1974)	Criminology
Combined	Homicide/assault	Negative	41 states + DC	2004–2009	Serial cross-sectional	Linear mixed-effect regression	Yes	$10,000 spent on social services (including education and welfare benefits, transportation, environment, public safety, and housing) and public health per person in poverty associated with 0.87 fewer homicides per 100,000 1 year later	Sipsma, H. (2017)	Health Policy and Management

^1^ Positive = higher spending associated with higher rates of violence. Negative = higher spending associated with lower rates of violence. Null = no statistically significant finding

Studies examined how government spending on social welfare (*n* = 21), health (*n* = 12), law enforcement (*n* = 8), education (*n* = 10), and/or community development (*n* = 2) was associated with homicide/assault (*n* = 21) and/or suicide (*n* = 14). Eleven studies explored more than one spending category and two explored both violence outcomes. Suicide was most often explored in relation to social welfare (*n* = 10) and health (*n* = 8) spending, whereas no studies explored the association between community development or law enforcement spending and suicide. Studies of suicide typically sourced mortality data from the Center for Disease Control (CDC) National Center for Health Statistics. Studies of homicide/assault most often measured rates of violent crime, including murder, rape, robbery, and aggravated assault, sourced from the FBI Uniform Crime Reporting (UCR) data. Five studies examined the impact of spending on firearm violence specifically, including firearm homicide rates, firearm suicide rates, school shootings, and injurious police shootings [46, 48, 52, 53, 60].

All studies were ecologic (with 1 at the national level, 19 at the state level, 5 at the county level, 5 at the city level, and 3 at the census tract level); 9 were cross-sectional, and 24 were serial cross-sectional. The most studied time periods were the 1990 s (*n* = 12) and early 2000 s (*n* = 11). Analytic approaches varied, with most studies (*n* = 28) employing multivariable models that adjusted for a range of confounders.

In total, 52 associations were examined in these 33 publications. The general direction of the study findings, by spending and violence types, is shown in Table 2. For example, of the 21 analyses of social welfare spending, 11 examined the outcome of homicide/assault, and six found that higher spending was associated with less homicide/assault, one found that higher spending was associated with more homicide/assault, and four reported null results. Six out of the ten analyses of social welfare spending and suicide found that more spending was associated with lower suicide rates; the others found a null association. Like social welfare, most papers exploring spending on health, education, and community development found increased spending associated with reductions in violence, followed by a substantial number of studies finding null associations. A few analyses, however, linked increased spending to higher rates of violence, including one analysis of health spending and suicide rates, and two analyses of education spending, one examining homicide/assault and the other suicide. There was less consistent evidence that increased law enforcement spending was associated with violence, with five out of seven studies finding null association and two finding reductions in homicide/assault.

**Table 2:** Synthesized findings of the direction of associations, by spending category and violence outcome

Violence outcome	Direction of association with increased spending1	Spending category					
		**Social welfare**	**Health**	**Education**	**Law enforcement**	**Community development**	**Combined (aggregate of all social services)**
**Homicide/assault(n=30)**		**(n=11)**	**(n=4)**	**(n=6)**	**(n=7)**	**(n=1)**	**(n=1)**
	Negative	6 (55%)	1 (25%)	3 (50%)	2 (29%)	1 (100%)	1 (100%)
	Positive	1 (9%)	-	1 (17%)	-	-	-
	Null	4 (36%)	3 (75%)	2 (33%)	5 (71%)	-	-
**Suicide(n=22)**		**(n=10)**	**(n=8)**	**(n=4)**	**(n=0)**	**(n=0)**	**(n=0)**
	Negative	6 (60%)	5 (63%)	2 (50%)	-	-	-
	Positive	-	1 (12%)	1 (25%)	-	-	-
	Null	4 (40%)	2 (25%)	1 (25%)	-	-	-

^1^ Positive=higher spending associated with higher rates of violence. Negative=higher spending associated with lower rates of violence. Null= no statistically significant finding

## Discussion

### State of the research

This scoping review examined if and how researchers have approached studying the association between government spending and interpersonal and self-directed violence, whether findings point towards consensus, and what gaps remain. We found that there is widespread and growing interest in understanding the ways that governments’ budget allocation relates to risk of interpersonal violence and suicide. Peer-reviewed publications on this topic date back to at least the 1970s, covering a range of disciplines including health, economics, and criminology. The breadth of perspectives underscores the nuanced and multidisciplinary nature of this research question and the interest many have in answering it. Further, while trends in publication on this topic have fluctuated over time (with 10 studies published in the 2000s and 6 published in the 2010s), there have already been 9 publications in just the first four years of the 2020s. This increase may reflect evolving perspectives on government approaches to safety. The 33 studies included in this review – despite variations in approach and findings – offer insights into whether and to what extent government spending may influence violence [12].

### Government spending and violence

Our assessment of the direction of associations and consistency of findings across studies provides a basis for identifying potential areas of consensus in the literature. Findings on the impact of increased spending on violence were mixed across all spending categories except for community development, for which only one study met inclusion criteria. This variation likely reflects the differences in study populations and methods, including strategies to account for the complexities of the temporal dynamics in public spending across different contexts. For instance, while law enforcement spending may yield short-term reductions in crime through increased enforcement, these benefits can be temporary and may be accompanied by negative consequences [69]. In contrast, spending in areas such as education or parks and recreation may have a delayed impact, indirectly reducing crime over time by expanding access to education and employment, improving neighborhood conditions, and providing positive outlets for youth engagement [70]. Similarly, social welfare spending may reduce the risk of violence in the short term by addressing immediate economic insecurity through measures like direct cash payments, while also offering long-term benefits by stabilizing households and improving neighborhood economic conditions [45]. Findings may therefore be expected to differ depending on whether and what duration of lags are modeled. Furthermore, government spending may not affect all people and places equally; studies have found variations in impact based on neighborhood advantage [36], city size [71], and sociodemographic characteristics like gender [57].

Overall, while some studies found null results and a few found unexpected positive associations, most studies assessing the impact of government investment in the social determinants of health – including health, social welfare, education, and community development – found that more spending was associated with less interpersonal violence and suicide. This finding aligns with the field’s understanding of the underlying drivers of violence [6], and with prior research connecting spending on these structural factors with improvements in other health outcomes [72, 73]. Reductions in homicide/assault were most often associated with social welfare spending, which is unsurprising considering prior work on poverty alleviation, social support programs, and community development initiatives on reducing crime and violence [6, 74]. For example, a recent review found income support policies were consistently protective against interpersonal firearm violence [14], and another found social protection policies and programs were largely protective against suicide [32]. Notably, we found that health spending may be a promising strategy to intervene on suicide – with 5 of the 8 studies assessing this relationship finding a protective association – which is supported by evidence suggesting close relationships between mental health services, crisis intervention, and suicide prevention.

Studies of law enforcement spending captured in this review reached less consensus, with more (*n* = 5) finding no association than protective associations (*n* = 2). This aligns with prior research showing no relationship between the number of police officers in a community and violence [75] and between police funding and homicide clearance rates across cities [76]. Although short-term declines in violence can occur due to deterrence or incarceration, policing can also generate fear, weaken social cohesion (an important protective factor against violence) and drive higher incarceration rates, which can ultimately reinforce cycles of violence by eroding human capital, limiting labor market opportunities, and increasing risk of further criminal legal system involvement [69, 77]. Further, policing, in some cases, may contribute to violence directly, with 1,769 people injured or killed by police in the US from 2015 to 2020, disproportionately in communities most affected by structural violence [60].

### Considerations for future research

Future research should leverage the groundwork established by these 33 publications, considering the analytic complexities inherent in researching this topic. While we did not restrict article inclusion based on analytic approach in this review, it is important to note that longitudinal and quasi-experimental designs are generally more rigorous than cross-sectional ones, and multivariable regression analyses have the potential to be much less biased by confounding compared to bivariate correlations or descriptive associations. Notably, researchers should carefully consider the potential causal structure for the context and relationships at hand and transparently communicate these assumptions. It is also important to consider the potential reciprocal relationship between spending and violence: funding allocations, particularly for certain categories like health and law enforcement, may be influenced by the pre-existing burden of violence, raising concerns about reverse causation. While lagging the dependent variable (violence) by at least one year, as done in many analyses included here, may help address this issue, future studies may consider using more advanced longitudinal causal inference methods, like g-computation, inverse probability of treatment weighting, and Longitudinal Targeted Maximum Likelihood Estimation, which allow researchers to account for time-varying confounding and obtain more accurate estimates of causal effects [78].

Limitations in availability of data may also lead to methodological concerns. All study designs captured in this review were ecologic, most often at the state level, which was somewhat expected given the contextual nature of the exposure and availability of outcome data. While state-level data may be easily accessible over multiple years, states are large and heterogenous, and aggregation may obscure important differences between places and increase the possibility of uncontrolled confounding [79]. Future research should explore sub-state heterogeneity, study the smallest and/or most theoretically relevant unit of analysis, and employ multi-level studies that examine contextual exposures and individual-level outcomes [80]. Additionally, studies would benefit from leveraging variation across both place and time, which would help mitigate threats to validity from time-invariant and time-varying confounding [81] while increasing power and generalizability compared to single-state or -city studies. Additionally, data for homicide/assault outcomes were most often sourced from the FBI UCR, which faces known limitations including underreporting, inconsistent data quality, and restrictions in scope, hindering its ability to provide a comprehensive understanding of violence [82]. The development of data systems to capture the full spectrum of nonfatal violent incidents, address reporting discrepancies across jurisdictions, and adapt to evolving forms of violence would ensure more accurate and actionable insights for effective intervention strategies.

Additional challenges in studying government spending and violence should also be considered. The isolated assessment of spending categories, as was done by most authors, may mask important inter-category dependence. For instance, education spending may have a more significant impact on reducing violence if social welfare spending is also high, given that communities may first require basic support before educational initiatives can thrive. This type of interdependency was addressed by one study that examined spending ratios in addition to absolute amounts, finding that, while neither police nor health spending alone was associated with reductions in violence, state-level overspending on police relative to health was linked to 46% higher victimization rates locally [60]. Future work should consider the interrelationships between spending categories and the implications of studying them in silos. Additionally, while our review was restricted to studies examining broad categories of spending, this aggregation may mask insights into how government funds within categories can be most effectively spent. For instance, the allocation of law enforcement funding, a sizable portion of many city and state budgets, is likely to carry strong implications for public safety. Some cities have more healing-centered tactics, like community-based violence intervention or mental health response teams, funded from the police budget, which we would expect to have a different effect on violence than other policing strategies, like those that place additional officers on the streets, as in the COPS program [75]. Future work should investigate how budgets within spending categories are allocated and whether these allocations affect the relationship between funding and violence.

Future research should also aim to fill gaps that remain underexplored. For example, despite the particularly high rate of firearm violence in the US – and potentially unique epidemiology and etiology of firearm violence compared with other forms of violence – we found that firearm violence was rarely examined as an outcome in studies of government spending. This is consistent with a recent review of income support policies and firearm violence, which identified only four studies globally on this topic [14], highlighting the pressing need for future research on the relationship between government budgets and firearm violence. Given research highlighting the violence-reducing potential of urban blight removal and greening [83, 84], further research into municipal expenditures on parks, recreation, and community development – of which we identified only one publication – is also warranted. Additionally, no studies explored the connection between law enforcement spending and suicide, but racialized and class-based differences in police encounters and risk of police brutality are associated with mental health [85] and suicide risk [86]. Finally, while our review was restricted to publications with quantitative results, none of the studies reviewed incorporated a qualitative component. Given the complexity of these research questions and inherent limitations of quantitative approaches alone [87], future studies should consider mixed methods designs.

While further research is needed to understand the pathways connecting budget decisions to safety and health and to inform appropriate prioritization of government spending, it is crucial to recognize that academic research should only play a part in decision-making around how to effectively move towards safer and healthier communities. Power should also rest with those who will experience the consequences of budget decisions. Therefore, the voices of community members closest to the issues should hold a stronger stake in decision-making regarding government spending, bolstered by research findings, through methods such as participatory budgeting [88].

## Conclusion

Government investments play a role in creating or withholding the conditions that foster safety and health, driving the distribution of the social determinants of health, and, in turn, of interpersonal violence and suicide in the US. Modification of government budget allocation has the capacity to reduce self- and other-directed violence. While differences in contextual factors, temporal dynamics, research design and analysis, and measurement approaches likely contributed to mixed findings, this review suggests that spending on upstream drivers of violence – specifically social welfare, health, and education – may be a sustainable path to safety, without the need to rely on reactive responses like law enforcement alone.

Given the historical and contemporary trends of municipalities increasing funding to law enforcement at the expense of investments in social determinants of health [12], these findings suggest a need to reassess the way budgets are allocated to promote public safety and health, with an eye towards longer-term potential. Especially in the current political moment – amid threatened and actual federal defunding of economic support programs, social and environmental justice initiatives, community-based violence intervention efforts, and related research – generating the political will for this shift may require a fundamental re-conceptualization of government funded public safety. Adoption of a broader collective definition of “public safety,” expanded beyond immediate physical protection, to include freedom from the very conditions that drive violence and insecurity – including structural violence and insecurities in food, housing, education, and employment [77] – could subsequently necessitate a reassessment of the government’s obligations, compelling investment in the conditions that foster true security for all [6, 15].

While the studies identified in this review provide a foundational starting point, strategic funding choices for interpersonal violence and suicide prevention require further research to deepen our understanding of these relationships and examine perspectives of key actors, including individuals most impacted by violence. In the current political climate, it is essential for scholars to advocate for a deeper understanding of how what we fund shapes health and safety at all levels of government. This includes federal and state budgets, but also – and perhaps most critically – local budgets, where autonomy may be greatest [12]. As more municipalities across the US explore innovative strategies to prevent and address violence, it is crucial that research continues to identify the optimal priorities for government investment, with a strong emphasis on centering community voices in these decisions.

## Data Availability

Not applicable.

## Appendix A: Databases and Search Terms

**Table:**

Database	Search term
PubMed	(federal[Title/Abstract] OR state*[Title/Abstract] OR city[Title/Abstract] OR cities[Title/Abstract] OR county[Title/Abstract] OR counties[Title/Abstract] OR municipal*[Title/Abstract] OR local[Title/Abstract] OR government[Title/Abstract]) AND (spend*[Title/Abstract] OR investment*[Title/Abstract] OR invest[Title/Abstract] OR funding[Title/Abstract] OR fund[Title/Abstract] OR funds[Title/Abstract] OR budget*[Title/Abstract] OR expenditure*[Title/Abstract] OR “social protection“[Title/Abstract] OR “social protections“[Title/Abstract] OR (“Resource Allocation“[Mesh Terms]) OR (economics[MeSH Terms])) AND (education*[Title/Abstract] OR health[Title/Abstract] OR police[Title/Abstract] OR policing[Title/Abstract] OR social[Title/Abstract] OR welfare[Title/Abstract] OR public[Title/Abstract] OR (“Health Care Economics and Organizations“[Mesh Terms]) OR (“Health Expenditures“[Mesh Terms]) OR (“Social Work“[Mesh Terms]) OR (Poverty[Mesh Terms]) OR (“Public Health/economics“[Mesh Terms]) OR (“Public Health Systems Research“[Mesh Terms]) OR (“Social Determinants of Health“[Mesh Terms])) AND (violen*[Title/Abstract] OR firearm*[Title/Abstract] OR gun[Title/Abstract] OR guns[Title/Abstract] OR gunshot*[Title/Abstract] OR homicide*[Title/Abstract] OR suicide*[Title/Abstract] OR (Homicide[Mesh Terms]) OR (“Wounds, Gunshot“[Mesh Terms]) OR (Suicide[Mesh Terms]) OR (Firearms[Mesh Terms]) OR (Violence[Mesh Terms])) NOT (butter[Title/Abstract]) Filters: Humans, English, Exclude preprints
Scopus	TITLE-ABS-KEY ((federal OR state* OR city OR cities OR county OR counties OR municipal* OR local OR government) AND (spend* OR investment* OR invest OR funding OR fund OR funds OR budget* OR expenditure* OR “social protection” OR “social protections”) AND (education* OR health OR police OR policing OR social OR welfare OR public) AND (violen* OR firearm* OR gun OR gun OR gunshot* OR homicide* OR suicide*) AND NOT (butter))
ProQuest search (Databases: PAIS Index, EconLit, ERIC, Social Services Abstracts, Sociological Abstracts, Black Studies Center)	Title, abstract, subject((federal OR state* OR city OR cities OR county OR counties OR municipal* OR local OR government) AND (spend* OR investment* OR invest OR funding OR fund OR funds OR budget* OR expenditure* OR “social protection” OR “social protections”) AND (education* OR health OR police OR policing OR social OR welfare OR public) AND (violen* OR firearm* OR gun OR guns OR gunshot* OR homicide* OR suicide*) NOT (butter))
Web of Science Core Collection	TS=((federal OR state* OR city OR cities OR county OR counties OR municipal* OR local OR government) AND (spend* OR investment* OR invest OR funding OR fund OR funds OR budget* OR expenditure* OR “social protection” OR “social protections”) AND (education* OR health OR police OR policing OR social OR welfare OR public) AND (violen* OR firearm* OR gun OR guns OR gunshot* OR homicide* OR suicide*) NOT (butter))
